# Destabilization of the TWIST1/E12 complex dimerization following the R154P point-mutation of TWIST1: an *in silico* approach

**DOI:** 10.1186/s12900-017-0076-x

**Published:** 2017-05-18

**Authors:** Charlotte Bouard, Raphael Terreux, Agnès Tissier, Laurent Jacqueroud, Arnaud Vigneron, Stéphane Ansieau, Alain Puisieux, Léa Payen

**Affiliations:** 1 Inserm UMR-S1052, Centre de Recherche en Cancérologie de Lyon, Lyon, 69373 France; 2 CNRS UMR5286, Centre de Recherche en Cancérologie de Lyon, Lyon, 69373 France; 3 LabEX DEVweCAN, Lyon, France; 4 UNIV UMR1052, Lyon, 69008 France; 5 Centre Léon Bérard, Lyon, 69373 France; 6 Université de Lyon1, ISPB, Lyon, 69008 France; 7 Laboratoire de Biochimie et Biologie Moléculaire (CHLS), Hospices Civils de Lyon, Lyon, 69003 France; 8 Pole Rhône-Alpes de Bioinformatique – Lyon Gerland (PRABI-LG), Lyon, 69007 France; 9 CNRS UMR 5305, Lyon, France; 10 Institut Universitaire de France, Paris, 75231 France

**Keywords:** TWIST1/E12, Dimerization, bHLH transcription factor, Molecular dynamics, *in silico* analysis

## Abstract

**Background:**

The bHLH transcription factor TWIST1 plays a key role in the embryonic development and in tumorigenesis. Some loss-of-function mutations of the *TWIST1* gene have been shown to cause an autosomal dominant craniosynostosis, known as the Saethre-Chotzen syndrome (SCS). Although the functional impacts of many *TWIST1* mutations have been experimentally reported, little is known on the molecular mechanisms underlying their loss-of-function. In a previous study, we highlighted the predictive value of *in silico* molecular dynamics (MD) simulations in deciphering the molecular function of TWIST1 residues.

**Results:**

Here, since the substitution of the arginine 154 amino acid by a glycine residue (R154G) is responsible for the SCS phenotype and the substitution of arginine 154 by a proline experimentally decreases the dimerizing ability of TWIST1, we investigated the molecular impact of this point mutation using MD approaches. Consistently, MD simulations highlighted a clear decrease in the stability of the α-helix during the dimerization of the mutated R154P TWIST1/E12 dimer compared to the wild-type TE complex, which was further confirmed in vitro using immunoassays.

**Conclusions:**

Our study demonstrates that MD simulations provide a structural explanation for the loss-of-function associated with the SCS TWIST1 mutation and provides a proof of concept of the predictive value of these MD simulations. This *in silico* methodology could be used to determine reliable pharmacophore sites, leading to the application of docking approaches in order to identify specific inhibitors of TWIST1 complexes.

**Electronic supplementary material:**

The online version of this article (doi:10.1186/s12900-017-0076-x) contains supplementary material, which is available to authorized users.

## Background

TWIST1 is a transcription factor belonging to the basic helix-loop-helix (bHLH) superfamily. During embryogenesis, it functions as an inducer of the epithelial-to-mesenchymal transition (EMT), a transdifferentiation process promoting the transient conversion of epithelial cells into mesenchymal cells [39, 41]. The TWIST1 protein is highly conserved from *Drosophila* to humans, where it plays a key role during embryonic morphogenesis, mesoderm patterning and development. It is involved in cell-type determination and differentiation during myogenesis, cardiogenesis, neurogenesis, hematopoiesis and osteogenesis [1, 26, 30]. The aberrant expression of TWIST1 has recently been implicated in cancer development, by fostering tumorigenesis and promoting the invasion-metastasis cascade [2, 17, 41].

One of the major functions of proteins belonging to the bHLH superfamily, which includes TWIST1, TWIST2, E12, E47, HAND1 and HAND2 [31, 38], is their interaction, once dimerized, with E-box sequences (CANNTG). TWIST1 proteins can form either homodimeric (TWIST1/TWIST1) or heterodimeric complexes, mainly in association with E2A proteins. This dimerization is a prerequisite for the recognition of E-box sequences, and involves basic domains and key residues of the bHLH domains [4, 29]. These complexes can bind to numerous variable canonical core recognition E-box sequences with different levels of affinity, and display distinct and sometimes even antagonistic functions during embryonic development [6, 7, 14, 24, 35]. The differences in the composition of the E-box sequences, in effect, largely modulate the in vivo function of cellular transactivation by closely related transcription factors [4, 5, 9, 16, 19].

**Fig. 1:**
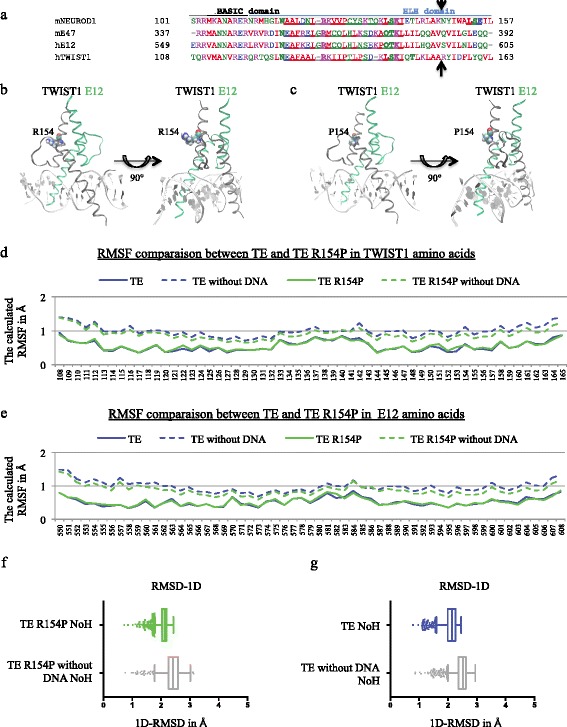
Comparison of the persistence of the wild-type TWIST1/E12 (TE) dimer and mutated R154P TE dimer with and without DNA. **a** Primary sequence alignment of the bHLH domains of the NEUROD1, TWIST1, and E2A proteins. Residues within the interhelical loops are underlined, h and m stand for human and murine, respectively. The arrows indicate the localization of the R154 residue and its equivalent on the other proteins. **b**-**c** 3D representation of the conserved TWIST1 (*grey ribbon*)/E12 (*green ribbon*) complex in the TE and TE R154P dimers in frontal (*left*) and lateral (*right*) views. The localization of the (**b**) arginine and the (**c**) proline 154 residues in the TWIST1/E12 and TWIST1/E12 R154P complexes on the TWIST1 ribbon is highlighted in CPK. **d**-**e** The root mean square fluctuation (RMSF) of TWIST1 and E12 amino acids were estimated during 10 ns *in silico* molecular dynamics (MD) simulations using the VMD 1.9.1 software. The graphical representation showed the calculated RMSF in angstroms (Å) for each (**d**) TWIST1 or (**e**) E12 residue in the mutated R154P TE model with (*green line*) or without (*dashed green line*) DNA anchor, and the TE model with (*blue line*) or without a DNA anchor (*dashed blue line*). **f**-**g** Box plots representing the root mean square deviation (1D-RMSD) by considering all of the atoms except hydrogen (NoH analysis) during 10 ns *in silico* molecular dynamics (MD) simulations. These distances were estimated during the MD simulations using the VMD 1.9.1 software (Graph of Labels Bonds). The NoH 1D-RMSD is shown in the (**f**) mutated R154P TE model with (*green line*) or without (*gray line*) DNA anchor, and (**g**) the TE model with (*blue line*) or without a DNA anchor (*gray line*)

The Saethre-Chotzen syndrome (SCS) is an autosomal dominant craniosynostosis characterized by facial and limb deformities caused by loss-of-function mutations of the *TWIST1* gene on chromosome 7p21 [25]. Over 160 *TWIST* mutations have been described in SCS patients, a majority of which are present in the bHLH domains, mediating protein dimerization, and involving a single base pair substitution (54% of mutations). These substitutions either create premature termination codons, which lead to truncated proteins, or substitute highly conserved residues in the bHLH region. These mutations affect the dimerizing ability and the DNA-binding properties of TWIST1. Deletions (25%) and insertions (15%) of nucleotides have also been reported in SCS patients. One such mutation, termed R154G, is the naturally-occurring, rare substitution of the arginine 154 amino acid with a glycine residue on the TWIST1 protein [33]. Arginine binds phosphate anions with a high affinity, and is often present in the active sites of proteins that bind phosphorylated substrates. As such, arginine residues play crucial roles in maintaining the overall balance of the charges within a protein. The Arginine residue plays a role in maintaining the overall charge balance of a protein. In addition, the NH_2_ groups of arginine are positioned so as to carry out H-bonds with partners or close residues of the protein, and strengthen the conformational structure of the dimer, while the biochemical properties of proline impede such an interaction. This arginine 154 residue is not conserved in other bHLH proteins (except for MYOD1 and TWIST2), which contain a glutamine, serine or asparagine residue at this position, residues that provide only limited physicochemical changes compared to an arginine residue. However, the R154 residue is instrumental in maintaining proper dimerizing functions of the TWIST protein, as demonstrated by Spicer et al. [37], who performed a proline point-mutation (*viz.* R154P) to ensure stronger physicochemical changes, since the lateral cycle usually disrupts the alpha helix of the HLH (Fig. 1a), and reported an impaired dimerization. This was later confirmed by our team in vitro in mammary epithelial cells by immunoprecipitation experiments [21].

Our present aim was thus to investigate whether our previously published *in silico* approach [3, 4] was able to predict the decrease in TWIST1/E12 dimerization observed in vitro and to decipher the molecular mechanisms involved, by comparing the dimerizing ability of the wild-type TWIST1/E12 (TE) complex with that of the mutated R154P TE (TE R154P) complex [21]. Furthermore, we examined whether these complexes bound to functional E-box sequences of targeted gene promoters using MD simulations, as previously described [3, 4]. This study was based both on the use of a recently published 3D dynamic structural model of the TE dimer [3, 4], initially generated using the murine NEUROD1/E47 crystal structure [3, 27], and on functional in vitro assays conducted on cancer cells to validate these computer-based models.

## Methods

### In silico analysis

#### Selection of X-ray structure and in silico model building

When initiating our study, we first selected the most appropriate 3D comparative model of the TWIST1/E12 complex from the structures available in the protein data bank (PDB)(Table 1). This choice mainly resided in the level of homology of these structures with TWIST1 and E12 and in the composition of the DNA sequence in the X-ray structure. Indeed, at the level of protein identity two candidates were identified, namely the human SCL/E47 complex (2YPB) and murine NEUROD1/E47 complex (2QL2), displaying 58% and 48% homology (at their conserved bHLH domain) with TWIST1, respectively, and 100% and 86% homology (at their conserved bHLH domain) with E12, respectively (Table 1). We next focused on the DNA sequence used in the generation of the model. Indeed, all four models were generated using a DNA sequence to stabilize the structures and facilitate the crystallization process. Having previously shown that the composition of DNA bases of the E-box plays a crucial role for DNA binding by bHLH dimers and for their transactivation activity [4], and that the E-box sequence (CATCTG) used to generate the NEUROD1/E47 structure is a highly specific E-box sequence of the TE complex [4, 9, 14], we finally selected this model for our study.

**Table 1:**
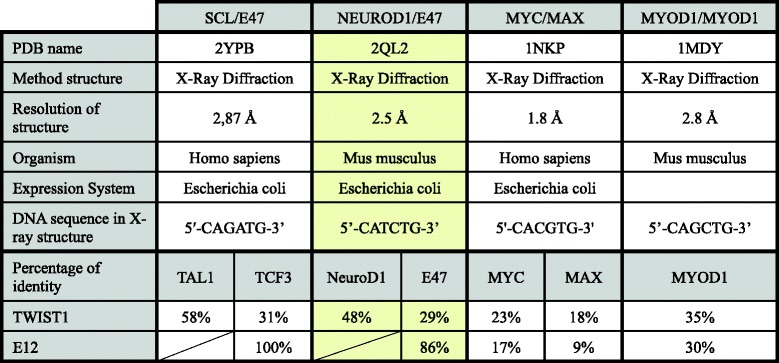
3D bHLH structures available in the protein data bank (PDB)

Table containing four protein complexes structurally close to TWIST1/E12 (TE). The information included in this table enabled us to select the most appropriate crystal structure, based primarily on the percentage of identity (TE), the DNA sequence in X-ray structure and the resolution of structure. The closest match (highlighted in yellow) was NEUROD1/E47

In effect, although the “CAGATG” E-box sequence contained in the SCL/E47 structure could be a potential DNA binding site of TWIST1, as predicted by De Masi et al. [9], and confirmed by EMSA experimentation in particular for the DNA binding of TWIST1/E12 dimer [5], the specificity of this box for TWIST1 homo- or heterodimers has not yet been determined. Lastly, the crystallization resolution of the NEUROD1/E47 structure was the highest between these two models at 2.5 Å.

The mouse NEUROD1 and E47 3D structure was thus obtained from PDB files, and the DNA sequence used to generate the model was kept in place, so as to preserve the proper position of the dimer in the DNA groove, and avoid altering residue-base interactions. Sequences, restricted to the bHLH domains, were aligned using the ClustalW 2.0 software (Fig. 1a). The alignments were then manually refined, taking residue properties (polarity, charge, hydrophobicity) into account. Two X-ray structures of the mouse NEUROD1/E47 complex have previously been reported (2QL2A/2QL2B; 2QL2C/2QL2D), which differ slightly (2D root mean square deviation 2D-RMSD: 0.618 Å). This divergence being fully corrected by a series of minimization steps (10,000) followed by 10 ns molecular dynamics (MD) simulations, the two models were considered equivalent, generating comparable homology models. Sequence alignments were submitted to the SWISS-MODEL Workspace to generate models of heterodimeric TWIST1 complexes by homology with the NEUROD1 template (PDB code 2QL2B)/E47 (PDB code 2QL2A). Minimizations (>10,000 steps) were carried out with the Sybyl-X 1.1 software package, using the Tripos method with the AMBER force field and a dielectric constant of 80 in order to refine and correct the positions of residue side chains. As mentioned above, the conserved TWIST1/E12 (TE) dimer model used throughout this study included a DNA sequence named the E-box (5′-TAGGC**CATCTG**GTCCT-3′) [3, 4, 27]. The R154P mutated TE complex was generated by substituting the arginine 154 residue by a proline residue, using the mutation function of the Sybyl-X 1.1 software package. The resulting *in silico* models studied herein were, therefore, the TE complex (original R154 residue) bound or not to the E-box sequence, as well as the R154P mutated (P154 residue instead of R154) TE complex bound or not to the E-box sequence. All of the models were generated following the same algorithm and using the same parameters.

### Molecular dynamics simulations

The established homology models were visualized with the VMD 1.9.1 software. The resulting model was inserted into a parapipedic TIP3P solvent box by means of the add solvation box module of the VMD 1.9.1 software. A distance of 15 Å was set between the surface of the protein and the limit of the solvent box.

Conditions were computed to reach neutral charges before adding sodium and chloride to concentrations corresponding to physiological conditions. The model was minimized with the NaMD 2.8 b1 software for 1000 steps and molecular dynamics simulations, and was computed on a 144 xeon core CPU cluster supercomputer (SGI Altix). Simulations were carried out at constant temperature (300 K) and pressure (1 atm) and by implementing the widely used CHARMM 27 force fields. The time frame was set at 1-fs and the Langevin and PME algorithms were applied. A conformation was sampled every 10-ps.

In the first instance, the effect of the R154P mutation of TWIST1 on the molecular interactions of the TE dimer with the E-box sequence was investigated in comparison with the effect of the WT TWIST1 protein, over a 10 ns experimental time-course. In the second instance, the impact of the anchor provided by the DNA sequence (E-box) on the binding efficacy of the WT or R154P mutated TE dimer was studied by conducting MD simulations over a time course of 10 ns.

### Definition of the domains studied

**Fig. 2:**
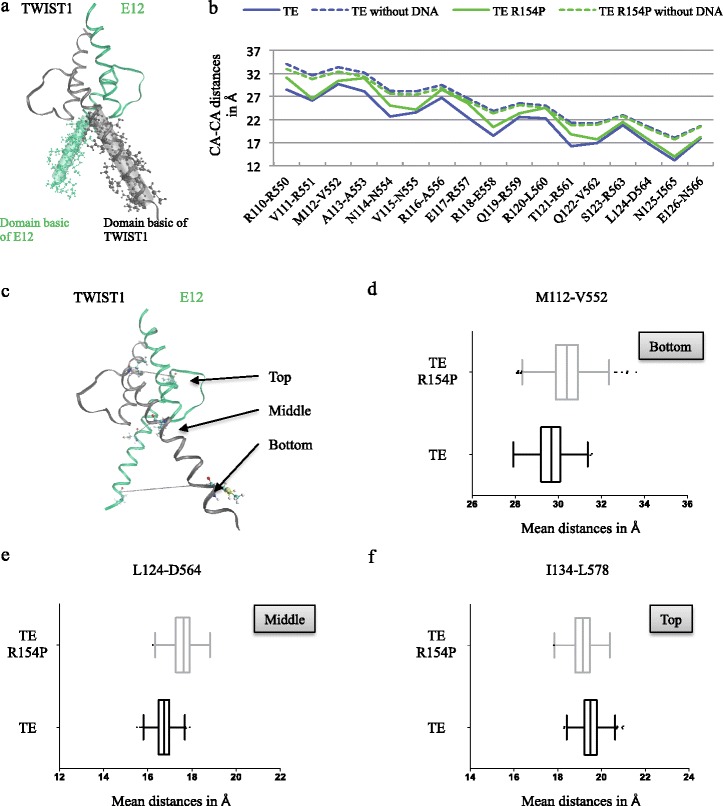
Comparison of the persistence of the wild-type TWIST1/E12 (TE) dimer and mutated R154P TE dimer. **a** 3D representation of the conserved TWIST1 (*grey ribbon*)/E12 (*green ribbon*) complex. Description of the position of the basic domain residues is represented as a cartoon and CPK in grey for TWIST1 (from R110 to E126) and in green for E12 (R550 to N566). **b** The CA-CA distances between pairs of basic domain residues on TWIST1 and E12 were estimated during 10 ns *in silico* molecular dynamics (MD) simulations using the VMD 1.9.1 software. The graphical representation shows the calculated mean distances between pairs of TWIST1/E12 residues in angstroms (Å) in the mutated R154P TE model with (*green line*) or without (*dashed green line*) DNA anchor, and the TE model with (*blue line*) or without a DNA anchor (*dashed blue line*). **c** 3D representation of the conserved TWIST1 (*grey ribbon*)/E12 (*green ribbon*) complex bound to DNA along with the E-box sequence (CATCTG), carried out using the VMD 1.9.1 software. The residues are numbered in each strand of the 5′-CATCTG-3′ E-box core. The three pairs of residues are shown, namely M112-V552, L124-D564, and I134-L578, which were located at the bottom, in the middle and at the top of the dimer, respectively. **d**-**f** Box plots representing the distances (Å) between pairs of residues of the dimer as a function of time (total time = 10 ns, 1 frame per 10 ps), during the TE (*black line*) and TE R154P (*grey line*) 10 ns *in silico* molecular dynamics (MD) simulations. These distances were estimated during the MD simulations using the VMD 1.9.1 software (Graph of Labels Bonds). The lengths of H-bond interactions were represented via GraphPad Prism 5: “smooth, differentiate or integrate curve, with 8 neighbors”. Interactions between the (**d**) M112 of TWIST1 and V552 of E12 residue pair, close to the bottom of the dimer, (**e**) L124 of TWIST1 and D564 of E12 residue pair, close to the middle of the dimer, and (**f**) I134 of TWIST1 and L578 of E12 residue pair, close to the top of the dimer

**Fig. 3:**
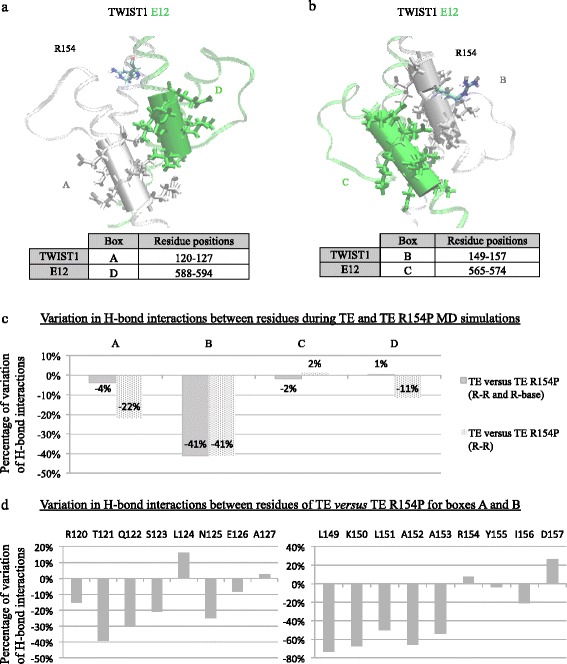
Consequences of impaired TWIST1/E12 (TE) dimerization on DNA binding. (**a**-**b**) 3D representation of the conserved TWIST1 (*grey ribbon*)/E12 (*green ribbon*) complex. Description of the position of the two series of residues (**a**) boxes A and D, and (**b**) B and C boxes, in the TE dimer are represented as a cartoon and CPK. The first dimerization blocs composed of boxes A and D (**a**), B and C (**b**) are represented by cylindrical grey and green solid surfaces, on the TWIST1 and E12 proteins, respectively. The localization of R154 on the TWIST1 ribbon is highlighted in CPK. **c** Evaluation of the impact of the R154P mutation on the number of H-bonds, in each individual box A to D by studing the percentage of variation of H-bond interactions between residues during TE and E R154P MD simulations, established between residue-residue and residue-DNA (R-R and R-base) or exclusively between residues (R-R). **d** Bar chart representing the variation of H-bond interactions between residues of the TE versus R154P TE complex. The sum of the interactions occurring between residues within the box A (*left*) and box B (*right*) of the wild type or mutated TWIST1 protein is highlighted. The percentage of cumulated occupancies of H-bond interactions occurring in the mutated R145P TE model is normalized against the TE model (100%). All cumulated occupancy values of the H-bonds were calculated as described in the Methods section. Briefly, H-bond interactions are assigned a value according to the distance between their atomic donors/acceptors during the 10 ns time-course of the MD simulation (interactions score 1 if their distance is under 2.1 Å, and 0 if above). Higher occupancy values being obtained for shorter and, therefore, more stable interactions. (SI function: SI(test_logic; value_if_true;value_if_false) with logic test:“<2.10”, value of 1 if true and 0 if false; NB.SI function: NB.SI(range;criterion))

Several domains were arbitrarily assigned to regions of the TE dimer in Figs. 2 and 3. Indeed, in Fig. 2a and b, we studied the position of the TE dimer in the DNA groove by studying the distance between basic domains of TWIST1 and E12 in the TE and TE R154P MD simulations. We considered that the basic domain of TWIST1 is composed of 17 amino acids: T108, Q109, R110, V111, M112, A113, N114, V115, R116, E117, R118, Q119, R120, T121, Q122, S123, L124, N125 and E126; whereas that of E12 is composed of the following 17 amino acids: E548, R550, R551, V552, A553, N554, N555, A556, R557, E558, R559, L560, R561, V562, R563, D564, I565 and N566. In the Fig. 2c, d, e and f, we defined three domains as “bottom”, “middle” and “top”. The former encompasses methionine 112 (M112) of TWIST1 and valine 552 (V552) of E12, localized at the bottom of TE dimer and near the DNA sequence, while “middle” designates residues leucine 124 (L124) of TWIST1 and aspartic acid 564 (D564) of E12, localized in the middle of TE dimer, and finally, the “top” is constituted of isoleucine 134 (I134) of TWIST1 and leucine 578 of E12 localized at the top of TE dimer.

Several domains were geographically defined in Fig. 3a and b, named A and B boxes for TWIST1, and C and D boxes for E12 (Fig. 3a), with A and D boxes and the B and C boxes facing each other, respectively (Fig. 3b). The residues composing A and B boxes of TWIST1 encompass amino acids “120–127” and “149–157”, respectively, whereas the composition in residues of C and D boxes of E12 contain amino acids “565–574” and “588–594”, respectively (see alignment Fig. 1a).

### The RMSF data interpretation

We studied the root mean square fluctuation (RMSF), which represents the flexibility of the protein model. We initially aligned the dimer with the VMD function RMSD trajectory tool (Extensions, Analysis, RMSD trajectory tool and align), and then calculated the RMSF with the VMD timeline function (Extensions, Analysis, timeline, calculate RMSF with window width (frames): 5 and step size (frames): 1). We obtained one RMSF per amino acid per frame and the average RMSF per amino acid was calculated using Microsoft Excel.

### The RMSD data interpretation

To complete RMSF analyses, 1D-root mean square deviation (1D-RMSD) calculations were carried out on all of the MD simulations, taking into account the constraints of the template as a reference. We obtained the 1D-RMSD with the VMD function RMSD trajectory tool (Extensions, Analysis, and RMSD trajectory tool). The 1D-RMSD calculations conducted encompassed all of the atoms, except hydrogen (analysis termed NoH). Before the extraction of 1D-RMSD values, we aligned all of the frames on the first frame by considering all of the proteins without hydrogen atoms.

### The DSSP data interpretation

We then used the online dictionary of secondary structure of proteins (DSSP) program http://swift.cmbi.ru.nl/gv/dssp/ to calculate DSSP parameters, such as secondary structure and solvent accessibility of protein residues. We analyzed the solvent accessibility value defined for each residue and we compared these values between the TE and TE R154P MD models.

### Variation of free energy of binding computed by mm-PBSA method

The resulting conformation of the protein DNA complex from molecular dynamics simulations with NaMD was extracted and several files were prepared using the Xleap module. The complex was neutralized with sodium counter ions. Ions and DNA were called the receptor and the protein the ligand. The complexe structure with ions was inserted into a TIP3P water PBC box. The amber 16 software was used with the FF14SB forcefield. The structure was minimized for 1000 steps and the gently thermalized with an increase temperature from 100 to 300 K in 60 ps to avoid destruction of interactions. The resulting structure was submitted to a 2 ns NTP molecular dynamics using a 300 K and 1 atm on Titan X GPU card. A frame was sampled every 20 ps so 100 frames were used for mm-PBSA calculations. For mm-PBSA calculation the calculation was performed with a total non-polar solvation term free energy modeled with one term (inp = 1), and with igb = 2.

### Alanine scanning method

This methodology computes variations in free energy by replacing each residue by an alanine residue. The alanine scanning calculation was performed with MOE from the Chemical Computing Group (CCG) company. The Amber10ETH forcefield was chosen, and starting from the resulting structure of the NaMD molecular dynamics, calculations were performed with Lowmode conformer generation (50 iterations). The interaction energy was computed for each conformation of each mutation with the DNA structure.

### Analysis of the persistence of the interactions arising during MD simulations

Among the interactions occurring between residue-residue (R-R) and residue-base (R-base), we focused our current investigation on hydrogen bond (H-bond) interactions established between donor and acceptor groups. The H-bond interaction is an intermolecular force involving a hydrogen atom and an electronegative atom, such as oxygen or nitrogen. H-bonds can be established between donor atoms and acceptor atoms of side chain residues. We studied the R-R or R-base interactions, which are established between atoms (O − H…:N; O − H…:O; N − H…:N; N − H…:O). The lengths of the H-bond interactions were represented via GraphPad Prism 5: “smooth, differentiate or integrate curve, with 8 neighbors”. These lengths were previously estimated around 2–2.8 Å depending on the nature of the acceptor and donor atoms [36]. Jeffrey categorizes H-bonds with donor-acceptor distances of 2.2–2.5 Å as “strong, mostly covalent”, 2.5–3.2 Å as “moderate, mostly electrostatic”, 3.2–4.0 Å as “weak, electrostatic” [22]. Consequently, we defined the threshold of the H-bond at 2.1 Å in order to mainly consider covalent H-bonds and to be more stringent. Using a computer software, we made the following hypothesis: any interaction occurring under this threshold was deemed “true” and assigned a score of 1, whereas any interaction occurring above this threshold was deemed “false” and given a score of 0 (SI function: SI(test_logic; value_if_true;value_if_false) with logic test:“<2.1”, value of 1 attributed if true and 0 if false; NB.SI function: NB.SI(range;criterion)). The scores were reckoned and the value obtained was designated as the rate of occupancy of H-bond interactions. Thus, for a given H-bond, the higher the rate of occupancy, the greater the number of interactions occurring under 2.1 Å, the higher the level of persistence of that H-bond during the MD simulation.

### In vitro analysis

#### Vector constructs for immunoprecipitation assays

The cDNA of TWIST1 (wild-type or R154P mutant) or wild-type cDNA of E12 were cloned into the pCI-neo expression vector with FLAG- or MYC-TAG, respectively. The constructs obtained were named FLAG-TWIST1 vector (T1), FLAG-TWIST1 R154P vector (T1 R154P), and MYC-TAG E12 vector (E12). The TWIST1 R154P mutant construct was modified by Genscript (T1 R154P). All final constructs were amplified using the MaxiPrep kit provided by PROMEGA and sequenced by Genoscreen compagny.

**Fig. 4:**
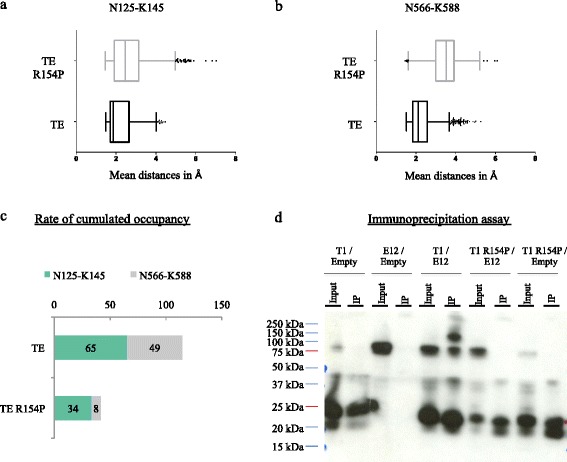
Consequences of impaired TWIST1/E12 (TE) dimerization on the loop structure of the bHLH domain. **a**-**b** Box plots representing the average distances (Å) between the OD1 atom of asparagine (N125) and the NZ1, NZ2 or NZ3 atoms of the lysine (K145) residues (**a**), and between N566 and K588 residues (**b**) during the wild-type TWIST1/E12 (TE) (*black line*) and mutated TE R154P (*grey line*) 10 ns *in silico* molecular dynamics (MD) simulations. **c** The horizontal bar chart shows the cumulated occupancy values for the H-bond interactions established between the N125-K145 residues of TWIST1 (*grey*) and the N566-K588 residues of E12 (*green*) during the TE and TE R154P MD simulations. All cumulated occupancy values of the H-bonds were calculated as described in the Methods section. Briefly, H-bond interactions are assigned a value according to the distance between their atomic donors/acceptors during the time (the 10 ns of the MD) (interactions score 1 if their distance is under 2.1 Å, and 0 if above). Higher occupancy values being obtained for shorter and, therefore, more stable interactions. (SI function: SI(test_logic; value_if_true;value_if_false) with logic test:“<2,10”, value of 1 if true and 0 if false; NB.SI function: NB.SI(range;criterion)). **d** Western blot showing the interaction between wild-type TWIST1 (T1) or T1 R154P and E12, as assessed by immunoprecipitation assays. TWIST1 or mutated TWIST1 R154P and E12 were transiently produced in HEK293T cells. The TWIST1 protein was immunoprecipitated with a monoclonal α-FLAG antibody and the presence of endogenous E12 protein in the immunoprecipitates (IP) was assessed. input 10%. The protein sizes were 99 kDa for the hetreodimer TE, 73 kDa for the E12 protein and 25 kDa for the TWIST1 protein

#### Vector constructs for Streptavidin/Biotin assays

**Fig. 5:**
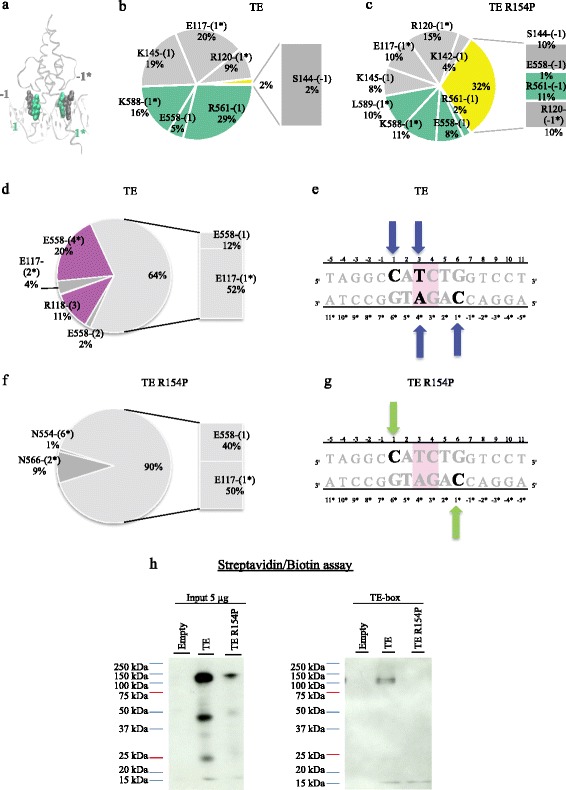
Consequences of impaired TWIST1/E12 (TE) dimerization on DNA binding. **a** 3D *in silico* representation of the TE complex bound to DNA, carried out using the VMD 1.9.1 software. The proximal flanking (−1; −1*) and first (1*; 1*) bases of the E-box are represented in grey and green VDW, respectively. **b**-**c** The pie charts show the percentages of cumulated occupancy of H-bonds established between residues of TWIST1 (*grey*) and E12 (*green*) and DNA bases during the (**b**) TE and (**c**) R154P TE MD simulations. Occupancies of H-bonds established with the proximal flanking (−1 and −1*) bases are represented in yellow, while the bar charts highlight the exact residues binding to those bases. The H-bonds established with the first consensus bases of the E-box (1 and 1*) are represented in grey and green for the TWIST1 and the E12 residues, respectively. **d**-**g** Distribution of “specific” H-bonds established between residues and E-box bases in the (**d**) TE and (**f**) mutated R154P TE molecular dynamics simulations. The pie charts show percentages of the total rate of cumulated occupancy of specific H-bonds established during the MD simulations. Consensus bases and variable bases of the E-box sequences are represented in grey and pink, respectively. The bar charts highlight the specific residues binding to cytosine (+1) and (+1*) bases, and show their implication in the DNA binding affinity. The DNA sequences are represented for (**e**) the TE and (**g**) mutated R154P TE dimers. The arrows highlight the major base interactions for the TE complex (*blue arrow*) and mutated R154P TE complex (*green arrow*). **h** Western blot showing that the tethered TWIST1/E12 R154P dimer (input left panel) was not able to interact with the TE-box unlike the tethered TWIST1/E12 wild-type dimer, as assessed by Streptavidin/Biotin assays. Tethered TE and TE R154P dimers were transiently produced in Hela cells. The protein sizes were 99 kDa for the thetered dimer TE and 25 kDa for the TWIST1 protein

The pBABE-neo expression vector was donated by H. Land & J. Morgenstern & B. Weinberg (Addgene plasmid # 1767) and was used as a negative control (named Empty in Fig. 5 h). The cDNA of the tethered TE complex composed of 896 amino acids, including a FLAG-TAG at the N-terminal position, TWIST1 cDNA, an amino acid linker and E12 cDNA, was cloned into the pBABE-neo expression vector [6]. This construct of the ectopic TE protein is approximately 99 kDa in size. The final construct was named the FLAG-TWIST1/E12 pBABE-neo expression vector (TE; Fig. 5h). This TE construct was modified by Genscript in order to obtained the tethered TE R154P mutant complex composed of 896 amino acids, including a FLAG-tag at the N-terminal position, TWIST1 cDNA with the substitution of the arginine 154 by a proline, an amino acid linker and E12 cDNA, was cloned into the pBABE-neo expression vector with the same size of TE (TE R154P; Fig. 5h).

#### Immunoprecipitation assays

HEK293T cells were provided by the ATCC. HEK293T and Hela cells were transfected in 10 cm in diameter culture dishes at a density of 80%–90% using the FuGENE HD Transfection Reagent (Promega #E2311) for 48 h. The transfection reagent contained 7.26 μg of the empty pCI-neo expression vector or of the pCI-neo expression vector containing cDNA coding the FLAG-TWIST1, FLAG-TWIST1 R154P, or MYC-E12 pCI-neo, as well as 10 μl of FuGENE. Following their transfection, cells were washed with cold PBS (2.7 mM KCl, 1.47 mM KH_2_PO_4_, 0.14 M NaCl, 3.4 mM Na_2_HPO_4_) and lysed in EBC solution (20 mM Tris pH 8.0, 385 mM NaCl, 2 mM EDTA, 3.5% NP40, PIC 1X, PMSF 1X). After incubation on ice for 20 min, lysates were collected by scrubbing the plates, sonicated (6 cycles of 10 s of ultrasound sonication with a 20 s gap between each cycle) and centrifuged for 20 min at 15,000 rpm at 4 °C. The supernatant was collected and the total protein content was quantified using the Bio-Rad Protein Assay (#500-0001).

After clearing by centrifugation, the protein extract was incubated with an anti-FLAG M2 resin (Sigma-Aldrich #A2220). After intensive washing to eliminate non-specific binding with EBC solution (20 mM Tris pH 8.0, 385 mM NaCl, 2 mM EDTA, 3.5% NP40, PIC 1X, PMSF 1X, without fetal serum), the resin was resuspended in Laemmli buffer and boiled for 5 min. After elimination of the beads by centrifugation, samples were reduced by adding β-mercaptoethanol and separated by SDS-PAGE. Proteins were analyzed with the rabbit polyclonal FLAG antibody (Thermo Fisher #PA1-984B) and the rabbit polyclonal V18 E2A antibody (Santacruz #sc-349) and a polyclonal goat anti-rabbit immunoglobulin conjugated with horseradish peroxidase as the secondary antibody (Dako #P0448). The detection was performed by Western blot analysis using the Luminol Reagent (Santa Cruz #sc-2048).

#### Streptavidin/Biotin binding assays

Hela cells were transfected in 6 cm in diameter culture dishes at a density of 80%–90% using the FuGENE HD Transfection Reagent (Promega #E2311) for 48 h. The transfection reagent contained 7.26 μg of the empty pBABE-neo expression vector or of the pBABE-neo expression vector coding the cDNA of the FLAG-TWIST1/E12 pBABE vector or FLAG-TWIST1/E12 R154P pBABE vector, as well as 21 μl of FuGENE reagent. Following their transfection, protein extractions were carried out as reported for the immunoprecipitation assay.

The Streptavidin/Biotin binding assay was conducted to isolate proteins (the TE complex) bound to specific DNA sequences (E-box sequences). To do so, total cellular proteins (extracted from cells transfected with the TE pBABE and TE R154P pBABE construct or with the empty pBABE control) were incubated with biotin-coupled DNA sequences, and the DNA bound TE complex was then purified by immunoprecipitation (IP) via the recognition of biotin by streptavidin beads. Hence, this approach decreased the risk of isolating non-specifically bound proteins. Briefly, both strands of DNA probes corresponding to various E-box sequences were labeled in their 3’ extremity with biotin. The probe used was the active TE-box (5′-CGTAGGCCATCTGGTCCTCG-3′). For probe hybridization, an initial denaturing step at 95 °C for 5 min was followed by a hybridization step at 57 °C for 10 min (the specific hybridizing temperature). Probe hybridizations were then confirmed on 3% agarose gels.

The binding between the DNA hybridized probes (3 μg) and the TE complex (80 μg) was carried out in binding buffer (100 mM KCl, 2% glycerol, 20 mM Hepes, 1 mM dithiothreitol and 0.1 mg/mL BSA) for 1 h at 30 °C. Biotinylated DNA probes bound to the TE complex were purified using DynaBeads® M-280 Streptavidin (Invitrogen #11205D). Coupled Streptavidin DynaBeads® bound to the DNA/TE complex (mutated or not) were run on a 12% SDS-polyacrylamide gel using a migration buffer (Tris glycine- SDS 1X) at 80 V for 10 min in the stacking gel, followed by the migration phase at 100 V for 120 min. The liquid transfer onto a PVDF membrane was conducted using a transfer buffer (Tris glycine 1X and 20% ethanol) at 200 mA for 80 min. The membrane was then saturated with 5% Tris-buffered saline (TBS) containing 0.05% Tween and 0.5% milk for 45 min, and incubated with the mouse monoclonal TWIST1 antibody (Abcam #ab50887) as a primary antibody, and a polyclonal rabbit anti-mouse immunoglobulin conjugated with horseradish peroxidase as the secondary antibody (Dako #P0260). The detection was performed by Western blot analysis using the Luminol Reagent (Santa Cruz #sc-2048).

## Results

The final 3D model used to generate our TE dimer was based on the *Mus musculus* NEUROD1/E47 complex (see Methods; Table 1). After aligning the four protein sequences using the ClustalW software [40], we mainly observed that NEUROD1 is composed of 357 amino acids with a bHLH domain containing amino acids 101–157, whereas TWIST1 is composed of 202 amino acids with a bHLH domain encompassing amino acids 108–164 (Fig. 1a). The crucial, strong physicochemical R154 residue of TWIST1 is a serine, an asparagine and a glutamine residue in E12, murine NEUROD1 and murine E47, respectively (Fig. 1a). As speculated by Spicer et al. [37], we could clearly observe that the substitution of the arginine residue with a proline residue, the lateral side chains of which contain a cycle known to destabilize the alpha helix, in this crucial position at the dimerization interface of TE altered the conformation of the dimer (Fig. 1b and c). Indeed, while the NH_2_ groups of arginine are positioned to carry out H-bonds with partners or close residues of the HLH loop, and strengthen the conformational structure of the dimer, the biochemical properties of proline impede such an interaction.

Next, to characterize the effect of this substitution on molecular mechanisms involved in the dimerization of the TE complex [3], we submitted the four *in silico* models (TE model with and without DNA, and TE R154P model with and without DNA) to 10 ns MD simulations. We then carried out root mean square fluctuation (RMSF) analyses to measure the structural flexibility of our proteins (Fig. 1d and e); the higher the RMSF value, the greater the flexibility. We observed an increase in RMSF values in the absence of DNA for TWIST1 and E12 residues, suggesting that the DNA sequence stabilizes the dimers. Nevertheless, no significant RMSF variation between TE and TE R154P MD simulations was observed, indicating the same level of flexibility of these two models (Fig. 1d and e). To complete this analysis, root mean square deviation (1D-RMSD) calculations were also carried out using the NoH parameter (all atoms were considered, except hydrogen atoms). Clearly, the presence of DNA during the dynamic simulations induced a decrease in 1D-RMSD values and enabled the stabilization of the dimer (Fig. 1f and g). Again, no significant difference could be observed between the TE and R154P TE models.

When focusing on R-R interactions between the 17 basic residues of TWIST1 and E12 (from R110 to E126 for TWIST1 and from R550 to N566 for E12) (Fig. 2a), the absence of DNA resulted in an increase in the mean R-R distances by almost 5 Å in TE and TE R154P molecular dynamics simulations (Fig. 2b). Since such a molecular instability in the DNA-free model may have overweighed the structural impact of the R154P point-mutation, or led to a biological artifact, all of the following MD simulations included a specific DNA E-box sequence [4].

We then evaluated the predictive value of our *in silico* strategy in assessing the functional disruption generated by the R154P mutation of TWIST1 on protein dimerization and DNA binding. Of note, protein dimerization precedes the binding of the complex to DNA sequences [13]. We observed that the mean distance between basic domains of TE R154P (solid green line) was greater than that of TE (solid blue line) (Fig. 2b). We then arbitrarily separated the dimer into 3 regions (see Methods), namely the “top” (M112-V552 distance), “middle” (L124-D564 distance) and “bottom” (I134-L578 distance) (Fig. 2c). A clear variation in distances between TWIST1 and E12 backbone residues (CA-CA interactions) was observed with the R154P mutation, indicating a distortion of the TE structure. Indeed, these distances either increased, namely between the M112 and V552 residues (Fig. 2d), and between L124 and D564 (Fig. 2e), or slightly decreased, namely between I134 and L578 residues (Fig. 2f). These results strongly suggest that the basic TE domains are further apart from the E-box sequence (positioned at the lower half of the dimer) during the mutated TE R154P MD simulation compared to the TE simulation, thus, impeding proper DNA binding (Fig. 2c, d, e and f). To complete these predictions, we calculated the dictionary of secondary structure of proteins (DSSP) parameter, which provides information on secondary structure and solvent accessibility of protein residues. No variation in the secondary structure prediction between TE and TE R154P for the arginine or proline residue were unveiled. Nevertheless, the R154P mutation TWIST1 decreased solvent accessibility of TWIST1 residues, mainly the loop residues of TWIST1 including residues I135 to L145. In contrast, this point-mutation did not modify the solvent accessibility of E12 residues (Additional file 1: Figure S1b and c).

Having shown a disruption in the CA-CA interactions between TWIST1 and E12 residues in the R154P mutated dimer, we investigated the effects of this mutation on the persistence of H-bonds (defined in the Methods as interactions < 2.1 Å between a hydrogen atom and an acceptor atom O, N) established between residue-residue (R-R) or residue-DNA base (R-base) during MD simulations (Fig. 3). We studied four dimerizing domains along the mutated complex, which are directly involved in dimerization and DNA binding, named A and B boxes for TWIST1, and C and D boxes for E12 (see Methods). A and D boxes (Fig. 3a) and B and C boxes (Fig. 3b) interacted with each other, respectively. As expected, the R154P mutation had the biggest impact on the persistence of H-bonds established by amino-acid residues situated within the B box, where the R154 residue resides, with a 41% decrease in H-bond establishment (Fig. 3c). When evaluating the persistence of H-bonds exclusively established between R-R, we observed that these were also impaired in the A box of TWIST1 (−22%) and D box of E12 (−11%). When we evaluated the interaction R-base, we observed an increase in these interactions, enabling a stabilization of the dimer on DNA. This clear decrease in the stability of these boxes, when comparing the TE and TE R154P models, confirms that DNA acts as an anchor for the C-α chain of HLH domains of TWIST1 (Fig. 3a, b and c). The destabilizing effect of the R154P substitution on TWIST1 was further explored, by studying the difference between the cumulated interactions of each residue within the A and B boxes of the wild-type and mutated TWIST1, during MD simulations. These simulations focused exclusively on the A and B boxes, since these domains were reported to experience major alterations (Fig. 3d). This strategy revealed that the impact of the R154P mutation is distributed throughout the α-helix. Moreover, the residues that presented the highest rate of variation in their H-bond interactions, such as threonine (T121), glutamine (Q122), and asparagine (N125) within the A box (Fig. 3d), and leucine (L149), lysine (K150), and alanine (A152) within the B box (Fig. 3d), were considered to be directly implicated in stabilizing the complex.

In addition, critical H-bonds localized in the heart of the dimer were disrupted, namely between the N125-K145 residue pair of TWIST1 and the N566-K588 residue pair of E12 in the R154P TWIST1 variant (Fig. 4a, b and c). This disruption occurs between the OD1 atom of the asparagine (N125 or N566) residues and either one of the three NZ1, NZ2 or NZ3 atoms of the lysine (K145 or K588) residues that are present under physiological conditions. Indeed, the interaction between the N566-K588 residue pair of E12 was largely impaired, revealing the clear impact of the TWIST1 mutation on the structure of heart of the TE complex, which is at the interface of the functional E-box recognition site (Fig. 4a, b and c). We confirmed the putative decrease in affinity by calculating the free energy of binding in kcal/mol between the TE complex and DNA using the mm-PBSA method. We obtained a dG score of −217 kcal/mol and of −191 kcal/mol in the TE wild-type and the TE R154P mutant, respectively. However, the dG computed with the mm-PBSA method implemented in Amber 16 software is different from the NAMD method used during our MD simulations. Consequently, we carried out the alanine scanning method, similarly to that used in our MD models. This AlaScan approach enabled us to define the affinity of TWIST1 for E12 and reciprocally of E12 for TWIST1 in the TE or TE R154P complexes. The affinity of TWIST1 for E12 was 46 kcal.mol^−1^ and 66 kcal.mol^−1^ in the TE complex and in the TE R154P complex, respectively. The affinity of E12 for TWIST1 was 55 kcal.mol^−1^ and 51 kcal.mol^−1^ in the TE complex and in TE R154P complex, respectively. Taken together, the AlaScan and mm-PBSA studies revealed that TWIST1 has a higher affinity for E12 in the TE complex, and thus confirmed that the R154P mutation may impact TWIST1 dimerization and its recognition or binding, once dimerized, to active E-box sequences. Consistently, we showed in vitro that TWIST1 R154P (T1 R154P) was not efficiently able to bind E12, as evidenced by immunoprecipitation assays (Fig. 4d). These assays revealed the presence of endogenous E12 protein (at 75 kDa, even under denaturing conditions) in the empty control lanes (T1 empty and T1 R154P empty) (Fig. 4d). Furthermore, although a clear TE dimer was obtained in the T1/E12 lane around 95–100 kDa, we also observed additional complexes T1/T1 (~50 kDa) and E12/E12 (~100–140 kDa), no dimer was observed in the T1 R154P/E12 lane (Fig. 4d).

Consequently, we explored the DNA binding affinity of the wild-type or mutated TE complexes, to investigate how such a mutation may affect canonical E-box recognition. Based on recent findings [4], we studied the particular involvement of the (1), (1*), (−1) and (−1*) DNA bases in the establishment of H-bond interactions between the wild-type or R154P mutated TE complex and the E-box sequence (Fig. 5a, b and c). We noted an important increase (+30%) in the implication of (−1) and (−1*) adjacent bases in the R154P variant (Fig. 5a, b and c). Moreover, this increase involved residues S144, R561 and R120 which were shown to play a pivotal role in the specific DNA binding affinity of the TE complex [3, 4] (Fig. 5b and c). Our previous findings demonstrated that the specific affinity of the TE complex to functional E-box sequences was associated with specific H-bond interactions (between atoms on the purine and pyrimidine nucleobases of the DNA and an atom of the side chain of TWIST1 or E12 residues contrary to the ‘non-specific’ H-bonds between oxygen elements on the phosphate groups of the DNA bases and an atom of the side chain of the protein residues) established with consensus bases (+1), (+2), (+2*) and (+1*), as well as variable central bases (+3), (+4), (+3*) and (+4*) of the E-box [4]. Here, we studied their distribution in the current MD simulations. We clearly noted that specific H-bond interactions were lost in the case of the mutated R154P TE complex, especially with variable central bases (Fig. 5d, e, f and g). This decrease was largely compensated by an increase (+26%) in specific H-bond interactions with consensus bases (+1) and (+1*) of the E-box sequences (Fig. 5d and f). In conclusion, these data strongly indicate that the R154P mutant, similarly to the TE complex binding to modified E-boxes [4], establishes alternative H-bond interactions with adjacent bases in our *in silico* models in an attempt to compensate for the decrease in structuration of the heterodimer. Hence, the R154P mutation not only affects the dimerization function but also the DNA binding function of the TE complex. This finding was validated in vitro using the Strepatvidin/Biotin assay. Indeed, although the tethered TE R154P dimer seemed less stable than the TE dimer (left panel), likely due to the impact of the R154P mutation on dimerization, it was unable to bind to the TE-box (Fig. 5h, right panel). Thus, the present *in silico* strategy is able to predict the impact of mutations occurring within the HLH domains of TWIST1, and possibly of E2A proteins, on the dimerization of the proteins and on DNA binding.

## Discussion

Our study provides yet another rationale for using molecular dynamics (MD) simulations as a predictive tool in the context of protein interactions, dimerization and DNA binding, and in determining the impact of protein point-mutations on the persistence of the HLH domains of the oncogenic TE complex [21]. Indeed, this approach previously enabled our team to study the effects of molecular modifications (insertions or single base mutations) of TWIST1 on the binding of the TE complex to DNA [3, 4, 28]. This *in silico* MD approach highlighted (i) the identification of the function of individual residues and molecular cooperation between residues, (ii) the predominant role of protein side chain residues, close to the heart of the complex, and those necessary for anchoring the dimer to DNA sequences, and (iii) the localization of the TE dimer on the DNA groove, by studying the shift towards adjacent E-box bases [4]. In agreement with Sauve’s model [34], we identified three different states of binding of the TE bHLH complex to functional and degenerate E-box sequences, based on MD simulations [4]. This *in silico* approach strongly suggested that we were able to define the molecular mechanisms implicated in the binding flexibility of the TE complex to E-box sequences of a targeted gene promoter, by predicting the proper transcriptional function.

Here, using a TWIST1 protein displaying the R154P mutation, we accurately predicted both an impairment in the protein dimerizing function and a subsequent decrease in the E-box binding affinity. We highlighted the determinant role of the interhelical loops in maintaining the structure of the TWIST1-DNA complex, by studying two variants with a 21-bp tandem repeat insertion in the TWIST1 gene, leading to the aberrant presence of seven extra amino-acids on positions 135 and 139 of the interhelical loop in SCS patients [12]. These insertions strongly modified the structure of the interhelical loops, and led to fewer contacts between interhelical loops and DNA. These proof of concept experiments were conducted prior to carrying out in vitro assays, namely immunoprecipitation and Streptavidin/Biotin assays, to validate the functional outcome of substituting residues.

Taken together, these results corroborate findings of Spicer et al. [37], who suggested that the SCS mutation R154P (helix I) led to a decrease in dimerization. This, in turn, may affect the quantity of functional TE complexes present in cells, explaining the intermediate loss of luciferase activity following the R154P mutation compared to the TE complex [21]. As we reported previously, this alteration impaired the transformation potential of the TE complex induced by the cooperation with the H-RAS^G12V^ protein [21].

This approach also provided a structural explanation for the loss-of-function associated with TWIST1-haploinsufficiency observed in SCS patients [3, 8, 18]. Using MD simulations and biochemical assays, we and others also highlighted the pivotal function of the R118H/Q/C, R120P, S144R, and K145E/Q residues in the molecular binding of the TE complex [4, 10–12, 18, 23, 28, 32, 33]. Mutations affecting these residues, as observed in SCS, lead to a decrease in the binding affinity of the dimer with the regulatory E-box sequences, and likely modify the transactivation functions of TWIST1 [12, 15, 20], resulting in an imbalance in the TWIST1/TWIST1 homodimer and in the TE heterodimer during the developmental process [6]. In the present work, we completed this study by showing *in silico* and in vitro that the H-bond integrity between residues of bHLH was partially impaired, which appears to be the major cause of the decrease in protein dimerization. To compensate for this, the R154P mutant alternatively establishes H-bond interactions with both non-specific and consensus bases of the E-box, thus modifying the conformation of the heart of the dimer by losing the crucial N125-K145 and N566-K588 H-bonds interactions. It is worth noting that K145 mutations are also reported in patients suffering from SCS syndrome [11].

Several bHLH proteins are known to dimerize with TWIST1, including the HAND proteins and proteins derived from the following genes: the TCF2/HEB, the TCF3/E2A (splice variants E12 and E47) and the TCF4/E2-2. Considering the degree of homology in their bHLH domains (92.7% identity with TCF2/HEB, 81.8% identity with TCF3/E2A E47 splice variant, and 89.1% identity with TCF4/E2-2), applying the current *in silico* approach to the evaluation of the dimerizing properties of all of these complexes would be highly relevant. In the future, it may be possible to identify specific transcriptional signatures according to the TWIST1 heterodimers generated, for example TWIST1/E-proteins versus TWIST1/TWIST1 or TWIST1/HANDs, and to their E-box binding specificities.

## Conclusions

These MD approaches allowed us to determine the key residues of TWIST1 and its partners involved in HLH dimerization, which may completely modify the balance of homo- and heterodimeric TWIST1 complexes. Overall, MD simulations may constitute a powerful tool to predict the biological impact of these alterations in a cancer cell, in terms of dimerization, but also of binding affinity to active E-box sequences.

## Additional file

Additional file 1: Figure S1. Consequence of impaired TWIST1/E12 (TE) dimerization on DNA binding. a-c: Dictionary of secondary structure of proteins (DSSP) parameters were calculated for the TE and TE R154P models, to obtain information on secondary structure and solvent accessibility of TWIST1 and E12 residues. (a) Bar chart presenting the variation in solvent accessibility of TWIST1 residues. (b) 3D representation of the conserved TWIST1 (grey ribbon)/E12 (green ribbon) complex displaying residue 154 represented in cartoon and residues impacted by DSSP calculations represented in CPK. (c) Bar chart presenting the variation in solvent accessibility of E12 residues. (PDF 196 kb)
