# Supplementary figures and images for: Destabilization of the TWIST1/E12 complex dimerization following the R154P point-mutation of TWIST1: an in silico approach
Source: BMC Struct Biol. 2017 May 18;17:6. doi: 10.1186/s12900-017-0076-x (PMC5437649; doi:10.1186/s12900-017-0076-x)

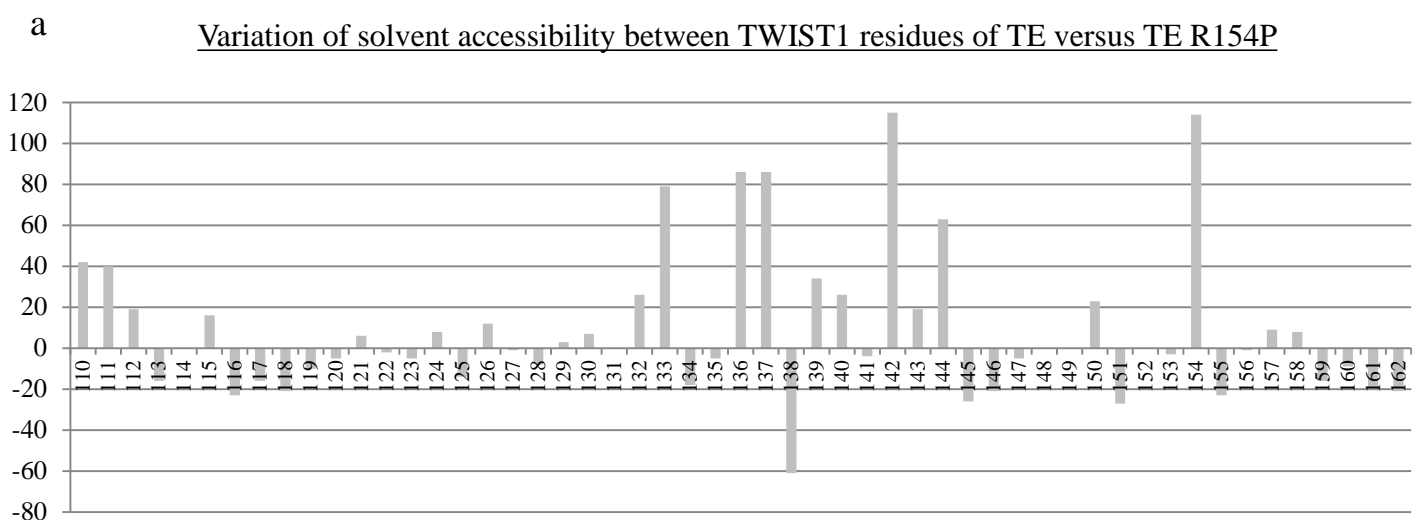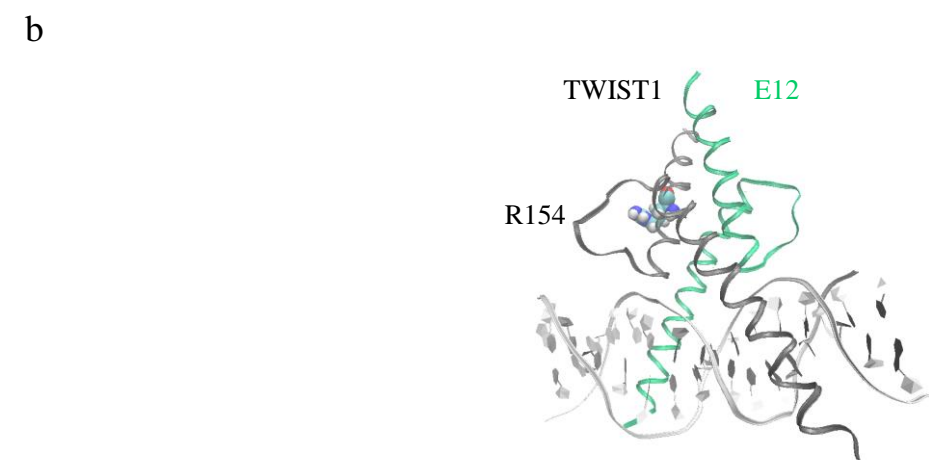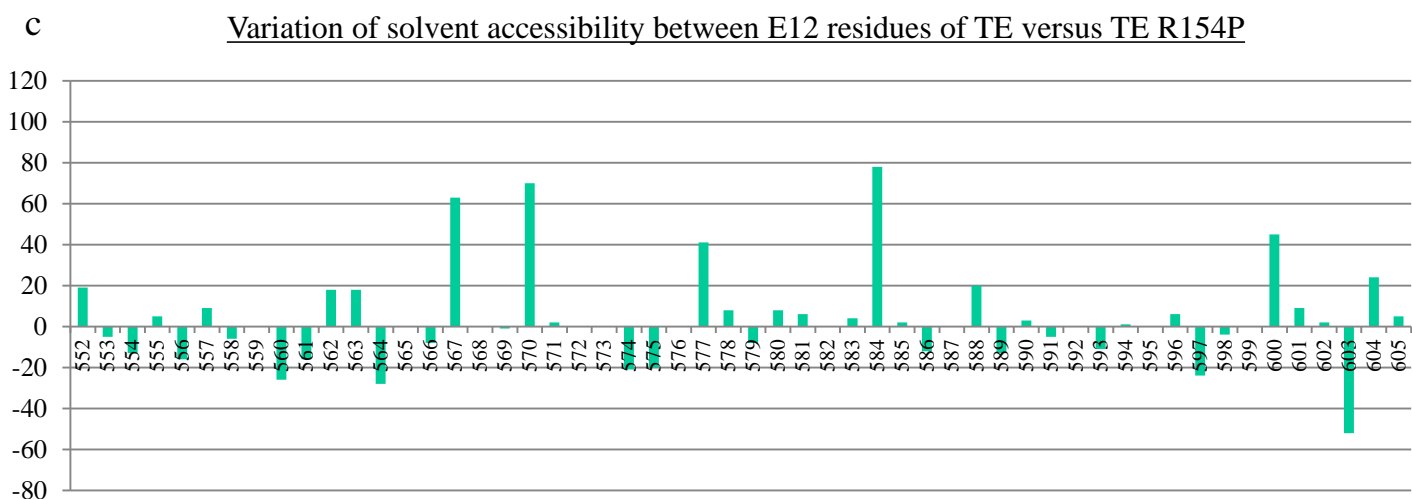

Figure S1

Supplement: Supplementary file 1 — Consequence of impaired TWIST1/E12 (TE) dimerization on DNA binding. a-c: Dictionary of secondary structure of proteins (DSSP) parameters were calculated for the TE and TE R154P models, to obtain information on secondary structure and solvent accessibility of TWIST1 and E12 residues. (a) Bar chart presenting the variation in solvent accessibility of TWIST1 residues. (b) 3D representation of the conserved TWIST1 (grey ribbon)/E12 (green ribbon) complex displaying residue 154 represented in cartoon and residues impacted by DSSP calculations represented in CPK. (c) Bar chart presenting the variation in solvent accessibility of E12 residues. (PDF 196 kb) [file 12900_2017_76_MOESM1_ESM.pdf]
